# All that glisters is not gold: a comparison of electronic monitoring versus filled prescriptions – an observational study

**DOI:** 10.1186/1472-6963-6-8

**Published:** 2006-02-10

**Authors:** Gwenn EC Wetzels, Patricia J Nelemans, Jan SAG Schouten, Boris LG van Wijk, Martin H Prins

**Affiliations:** 1 Department of Epidemiology, University of Maastricht, Maastricht, The Netherlands; 2 Department of Ophthalmology, University Hospital Maastricht, Maastricht, The Netherlands; 3 Department of Pharmacoepidemiology and Pharmacotherapy, Utrecht Institute of Pharmaceutical Sciences, Utrecht, The Netherlands

## Abstract

**Background:**

Poor compliance with antihypertensive medication is assumed to be an important reason for unsatisfactory control of blood pressure. Poor compliance is difficult to detect. Each method of measuring compliance has its own strengths and weaknesses.

The aim of the present study was to compare patient compliance with antihypertensive drugs as measured by two methods, electronic monitoring versus refill compliance.

**Methods:**

161 patients with a diagnosis of hypertension for at least a year prior to inclusion, and inadequate blood pressure control (systolic blood pressure ≥ 160 mmHg and/or diastolic blood pressure ≥ 95 mmHg) despite the use of antihypertensive drugs, were included. Patients' pharmacy records from 12 months prior to inclusion were obtained. Refill compliance was calculated as the number of days for which the pills were prescribed divided by the total number of days in this period. After inclusion compliance was measured with an electronic monitor that records time and date of each opening of the pillbox. Agreement between both compliance measures was calculated using Spearman's correlation coefficient and Cohen's kappa coefficient.

**Results:**

There was very little agreement between the two measures. Whereas refill compliance showed a large range of values, compliance as measured by electronic monitoring was high in almost all patients with estimates between 90% and 100%. Cohen's kappa coefficient was 0.005.

**Conclusion:**

While electronic monitoring is often considered to be the gold standard for compliance measurements, our results suggest that a short-term electronic monitoring period with the patient being aware of electronic monitoring is probably insufficient to obtain valid compliance data. We conclude that there is a strong need for more studies that explore the effect of electronic monitoring on patient's compliance.

## Background

Lack of compliance with antihypertensive drug regimens is assumed to be a major cause of failure to achieve adequate blood pressure control [1-3]. It is estimated that that at least 50% of the patients in a general hypertensive population do not take their antihypertensive medication as prescribed [4]. Improving compliance with prescribed drug regimens therefore remains a major challenge to the physician and it is extremely important to have accurate measures of compliance.

There is a continuing debate with regard to measurement of compliance, which is notoriously difficult. An ideal method to measure compliance should be valid and reliable. It should prove ingestion of the medication and give information about the timing of ingestion. Also the patient should not be aware of the compliance measurements and not be able to consciously influence the results [5,6].

At present, electronic monitoring is considered to be the new "gold standard"[7]. Electronic monitors are normal pill-bottles with a special cap that contains a microchip that registers time and date of every time that the bottle is opened. This method is more sensitive for detecting inadequate compliance than other methods, such as self-reports. However, its costs and other practical issues limit the use of electronic monitors in routine clinical practice.

Another method to assess compliance is the use of pharmacy records. Pharmacy refill records provide objective, unobtrusive and inexpensive estimates of compliance. Refill records of computerized pharmacy records are used increasingly as a source of compliance information [8]. Because refill compliance data only give information about whether or not the medication is obtained by the patient, it provides an upper bound for medication consumption. It allows identification of those patients that cannot be compliant simply because they do not obtain enough medication [8]. It is therefore to be expected that the actual proportion of patients with poor compliance is higher. To evaluate the value of using pharmacy records to identify poor compliers, it would be useful to compare the use of pharmacy records to determine compliance with a more sensitive measurement tool, such as electronic monitoring. However, comparisons of refill compliance with other objective compliance measurement methods within the same population are scarce.

The aim of the present study was therefore to compare compliance with antihypertensive drugs assessed by both refill compliance and electronic monitoring, within a population of patients with mild hypertension and to evaluate the agreement between these two compliance measures.

## Methods

### Study population

Patients were recruited from general practices in the South of the Netherlands. Patients were eligible if they met all following criteria: a) they had a diagnosis of hypertension, b) they had inadequate blood pressure control despite the use of antihypertensive medication and c) treatment escalation seemed appropriate. Treatment escalation was defined as increase in dosage, addition or change of antihypertensive drugs. Hypertension was, according to the national guideline of the Dutch College of General Practitioners (update 1999) [9], defined as a systolic blood pressure (SBP) ≥ 160 mmHg and/or diastolic blood pressure (DBP) ≥ 95 mmHg. All patients gave written informed consent. The Ethical Committee of the Maastricht University approved of the study.

### Electronic monitoring

Compliance with antihypertensive medication was measured by electronic monitoring during two months after inclusion. Medication was packaged in electronic pillboxes (Medication Event Monitoring System (MEMS), Aardex corp. Geneva). A microprocessor in the bottle cap registered the date and time of each opening. Each monitor was coded with an identification number that identified the study participant. At the end of the study, all data collected by the microprocessor were analyzed using PowerView version 2 software (Aardex corp. Geneva). Each opening was considered as being a single dose intake. Participants were informed that the MEMS monitor recorded the date and time of each opening of the medication bottle. They were instructed to keep their medication in the monitoring vial, to use no other source of anti-hypertensive medication and to remove only one dose at a time. Patients received a MEMS-monitor for each antihypertensive drug they used. Monitoring compliance was expressed as taking compliance = (total number of doses taken)/(total number of monitored days) × 100%. Patients were considered to be compliant if taking compliance was ≥ 85% on each antihypertensive drug they used.

### Filled prescriptions

Patients' records from computerized pharmacy systems were obtained. These records included the names of the prescribed antihypertensive drug(s), dosage, the quantity dispensed at each pharmacy fill and the dates of prescription fills. Refill compliance was determined from these pharmacy records during twelve months before the start of electronic monitoring.

Refill compliance during the interval between two prescription fills was calculated as the number of days for which the pills were prescribed divided by the total number of days in this interval. Only those medications that were filled at least three times during the twelve months were taken into account, so refill compliance could be calculated for at least two intervals. For each antihypertensive drug average refill compliance was computed by summing the refill compliance in each interval and dividing the sum by the number of intervals. Patients were considered to be compliant if average refill compliance was ≥ 85% on each antihypertensive drug they used.

### Statistical analysis

First, monitoring compliance (taking compliance) was plotted against refill compliance in a scatter diagram. Agreement between the compliance measures on a continuous scale was calculated using Spearman's correlation coefficient. Second, patients were classified into two categories: adequate compliers versus poor compliers. For both refill compliance and taking compliance patients were considered to be poor complier if they took less than 85% of any prescribed antihypertensive drug. Agreement between dichotomized refill and taking compliance was assessed using overall proportion of agreement, positive agreement, negative agreement and Cohen's kappa coefficient, which corrects for chance agreement [10]. In additional analyses, Cohen's kappa coefficient was determined using different cut-off points for compliance, namely 80%, 90% and 95%.

## Results

Both monitoring and refill compliance data were available for 146 of 161 patients who participated in the study. Two patients had to be excluded from the analysis since they did not follow the instructions on the use of the electronic monitor. Furthermore, the data from two monitors could not be retrieved due to a technical error. Finally, from eleven patients' pharmacy records were not available mainly because the patients moved to other regions. The baseline characteristics of the patients included in the analysis are shown in Table 1.

### Compliance with antihypertensive medication

Mean taking compliance as measured by electronic monitoring was 96.8 ± 12.1%. Five patients (3.4%) were considered as poor compliers, because taking compliance of at least one of their prescribed medications was below 85%. Average compliance in the group of poor compliers was 59.8 ± 23.9% (p < 0.001).

Mean refill compliance was 108.9 ± 28.3%, indicating stockpiling of medication by patients. Twenty-seven patients (18.4%) were identified as poor compliers, because refill compliance of at least one of their medications was below 85%. Average refill compliance in these patients was 86.2 ± 18.6% compared to 114.2 ± 26% in patients with adequate compliance (p < 0.001). From those 27 patients considered as poor compliers, 19 were prescribed more than one antihypertensive drug. In only four of these patients compliance was not satisfactory (<85%) for each prescribed antihypertensive drug.

### Agreement between electronic monitoring and filled prescription

**Figure 1:**
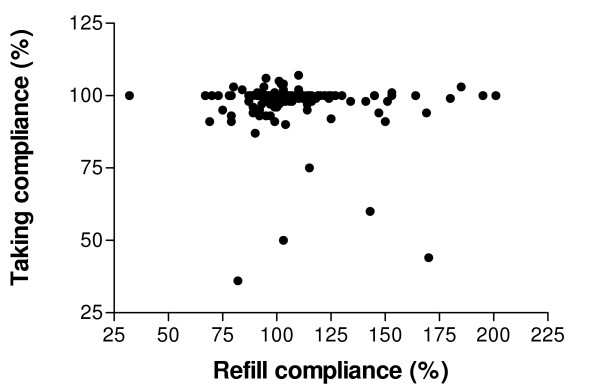
Comparison of refill compliance versus electronic monitoring (taking compliance).

Figure 1 presents a scatter plot for all patients of refill compliance (on the X-axis) versus taking compliance (on the Y-axis). There was very little agreement between the two measures and whereas refill compliance showed a large range of values, compliance as measured by electronic monitoring was high in almost all patients with estimates between 90% and 100%. The Spearman's correlation coefficient was -0.02 (95% CI: [-0.18-0.15]). After classification of patients into adequate compliers versus poor compliers, the agreement between refill compliance and taking compliance was also poor (Table 2). The overall proportion of agreement was 0.8. The proportion of negative agreement, which is the proportion of patients that are identified as complier by both methods, was 0.89. However, the proportion of positive agreement, defined as the proportion of patients that were identified as poor complier by both methods, was only 0.06. Cohen's kappa coefficient, which corrects for chance, was 0.005 indicating a very poor agreement between the two methods to determine compliance. Also when different cut-off points for compliance were used, the agreement between the two methods was poor. Using cut-off points of 80%, 90% and 95%, Cohen's kappa was -0.056, 0.020 and 0.009 respectively (table 3).

## Discussion

This study demonstrates that there is a poor agreement between compliance measured by electronic monitoring and compliance determined by filled prescription data within the same population of hypertensive patients. A remarkable finding is that many patients, who according to refill compliance data are not compliant with antihypertensive medication, are not identified as such by electronic monitoring which is considered to be the "gold standard". With electronic monitoring, less than four percent of the patients could be identified as poor complier. Using refill data, the percentage of patients with inadequate compliance was 18.5 percent. Determination of compliance based on filled prescriptions was expected to be a rather insensitive method to measure compliance. Only a part of the poor compliers can be identified using this method, because obtaining the medication is no guarantee that the patient actually takes them. Therefore refill compliance is expected to identify the minimum proportion of poor compliers within a population. Despite this fact, the use of refill data identified a higher percentage of patients with inadequate compliance than electronic monitoring. Several explanations may account for these results.

First, it may be argued that the proportion of poor compliers identified with electronic monitoring would be much higher when a more stringent measure to define compliance, such as correct dosing, was used. Correct dosing reflects the percentage of days on which the medication is taken as prescribed and is therefore much more sensitive for deviations from the prescribed regimen than taking compliance data. However, when this measure was applied to our population only six instead of five patients were identified as poor complier.

Second, it is possible that electronic monitoring in itself improves compliance because patients are aware that their medication behavior is being monitored. This awareness may have encouraged them to be more compliant than they used to be before the start of electronic monitoring. The hypothesis that electronic monitoring in itself improves compliance, is in agreement with the findings in other studies, which also point in this direction. First, most recent studies using electronic monitoring very often report relatively low proportions of patients with poor compliance [11-18] in contrast with the general assumption that 40 to 50% of the patients do not take their antihypertensive medication as prescribed. Second, Burnier et al. [14] demonstrated that a two-month period of monitoring of compliance was associated with a significant improvement of blood pressure, most likely resulting from increased compliance with antihypertensive drug therapy. Also Waeber et al. [12] found that three-month period of monitoring was associated with a significant improved control of blood pressure.

It can be concluded that the findings in our study stress the importance of the conditions that need to be met before electronic monitoring can be used to obtain accurate compliance data. First, it seems extremely important to conceal the purpose of the monitor from the patient to obtain valid compliance data, but this is really hard to accomplish in practice. Kruse and Weber [19] found a compliance of 91% in informed individuals compared to 78% in a group who did not understand the value of the electronic monitor. Second, studies with a monitoring period of at least 6 months demonstrate a clear-cut decrease in compliance over time suggesting that the effect of awareness of being monitored wanes over time [20-22]. From these studies it can be concluded that it is important to monitor compliance for at least six months in order to obtain valid measures of compliance [23].

Some methodological issues deserve further attention. First, the time periods wherein refill and taking compliance were measured, differed. The short duration of electronic monitoring (two months) and low refilling frequency during this time precluded stable estimation of refill compliance during the monitoring period itself. In this respect the present study is comparable to the study performed by Choo et al[24] which found moderate agreement (r = 0.32) between compliance measured during a three month monitoring period and refill compliance in the twelve months before electronic monitoring. The additional value of the present study is that it gives information about the proportion of patients with inadequate compliance instead of mean values of compliance. Whereas mean values give the impression that refill compliance is higher than monitoring compliance, proportions indicate that it is the other way around: in comparison with electronic monitoring, refill compliance measurements trace more poor compliers.

Second, in the primary analysis a cut-off point for compliance of 85% was used. It is accepted that patients are considered adherent with the prescribed medication regimen when they take 80–90% of their medicines [25-27]. However, historically used cut-off points are in many cases meaningless as some drugs are much more "forgiving" than others in term of missed dosing and the timing of ingestion [28]. The answer to the question "how much compliance is enough" requires knowledge of the pharmacokinetic and pharmacodynamic properties, which vary between antihypertensive drugs and also vary between individuals. At this stage we do not really know what level of compliance is necessary for individual antihypertensive medications. To undermine this problem Cohen's kappa was determined for different cut-off points. The agreement between both methods was poor, irrespective of the used cut-off point.

## Conclusion

To manage to problem of hypertension there is a need for accurate methods to measure compliance with medication. Unrecognized compliance problems may result in uncontrolled hypertension, unnecessary medication-switches and even hospitalization. It can be concluded that a short-term electronic monitoring period with the patient being aware of electronic monitoring, is probably insufficient to obtain valid compliance data. Electronic monitoring may however be a very useful tool to improve compliance by giving patients insight into their own dosing history. We conclude that there is a strong need for more studies that explore the effect of electronic monitoring on patient's compliance.

## Competing interests

The author(s) declare that they have no competing interests.

## Authors' contributions

GW supervised the study, was responsible for the data acquisition, data analysis and statistical analysis. PN was responsible for protocol development, and contributed to the statistical analysis and manuscript preparation. BvW helped to collect data and contributed to the manuscript preparation. JS and MP revised the manuscript. All authors read and approved of the final manuscript

## Pre-publication history

The pre-publication history for this paper can be accessed here:



## Figures and Tables

**Table 1:** Demographic and clinical characteristics of included patients (n = 146)

**Demographic characteristics**^**# **^(%)	
Age	
≤ 55	21
56–65	44
66–75	26
≥ 75	8
Gender	
Male	48
Female	52
Education	
Low	22
Middle	32
High	33
Very high	11
Employed	
Yes	31
No	68

**Clinical characteristics **(Mean (SD))	

Years with hypertension	8.5 (8.8)
SBP at inclusion	168.4 (16.3)
DBP at inclusion	95.4 (9.8)
Heart rate	73.2 (12.2)
BMI (kg/m2)	28.6 (5.6)
Nr. of prescribed antihypertensive drugs (%)	
1	45
2	41
3	11
> 3	2
Medication regimen (%)	
Once daily	84
Twice daily	13
> twice daily	3

# Some categories do not add up to a 100% because data were not available for all patients

**Table 2:** Refill compliance versus electronic monitoring (taking compliance)

		Taking compliance		
		**Adequate compliers**	**Poor compliers**	*Total*

Refill compliance	**Adequate compliers**	**115**	**4**	*119*
	**Poor compliers**	**26**	**1**	*27*
	*Total*	*141*	*5*	*146*

**Table 3:** Percentage of poor compliers and Cohen's kappa coefficients according to different cut-off points

**Cut-off points**		**80%**	**85%**	**90%**	**95%**
**% Poor compliance**	**Taking compliance**	3.4	3.4	4.8	14.4
	**Refill compliance**	11.6	18.5	23.3	32.2
**Cohen's kappa coefficient**		-0.056	0.005	0.020	0.009

## References

[B1] Mar J, Rodriguez-Artalejo F (2001). Which is more important for the efficiency of hypertension treatment: hypertension stage, type of drug or therapeutic compliance?. J Hypertens.

[B2] O'Rorke JE, Richardson WS (2001). Evidence based management of hypertension: What to do when blood pressure is difficult to control. Bmj.

[B3] Urquhart J (1994). Partial compliance in cardiovascular disease: risk implications. Br J Clin Pract Suppl.

[B4] WHO (2003). Adherence to long-term therapies: evidence for action. WHO.

[B5] De Klerk E, Vingerhoets A (2001). Measurement of patient compliance on drug therapy: an overview. Advances in behavioral medicine assesment.

[B6] Rudd P (1979). In search of the gold standard for compliance measurement. Arch Intern Med.

[B7] Cramer JA (1995). Microelectronic systems for monitoring and enhancing patient compliance with medication regimens. Drugs.

[B8] Steiner JF, Prochazka AV (1997). The assessment of refill compliance using pharmacy records: methods, validity, and applications. J Clin Epidemiol.

[B9] Walma EP, Grundmeyer HGJM, Thomas S, Prins A, Hoogen JPH, Laan JR (1999). NHG-standaard Hypertensie. Huisarts Wet.

[B10] Kundel HL, Polansky MP (2003). Measurement of observer agreement. Radiology.

[B11] Mallion JM, Dutrey-Dupagne C, Vaur L, Genes N, Renault M, Elkik F, Baguet P, Boutelant S (1996). Benefits of electronic pillboxes in evaluating treatment compliance of patients with mild to moderate hypertension. J Hypertens.

[B12] Waeber B, Vetter W, Darioli R, Keller U, Brunner HR (1999). Improved blood pressure control by monitoring compliance with antihypertensive therapy. Int J Clin Pract.

[B13] Weidler D, Wallin JD, Cook E, Dillard D, Lewin A (1992). Transdermal clonidine as an adjunct to enalapril: an evaluation of efficacy and patient compliance. J Clin Pharmacol.

[B14] Burnier M, Schneider MP, Chiolero A, Stubi CL, Brunner HR (2001). Electronic compliance monitoring in resistant hypertension: the basis for rational therapeutic decisions. J Hypertens.

[B15] Eisen SA, Miller DK, Woodward RS, Spitznagel E, Przybeck TR (1990). The effect of prescribed daily dose frequency on patient medication compliance. Arch Intern Med.

[B16] Waeber B, Brunner HR, Metry JM (1997). Compliance with antihypertensive treatment: implications for practice. Blood Press.

[B17] Leenen FH, Wilson TW, Bolli P, Larochelle P, Myers M, Handa SP, Boileau G, Tanner J (1997). Patterns of compliance with once versus twice daily antihypertensive drug therapy in primary care: a randomized clinical trial using electronic monitoring. Can J Cardiol.

[B18] Nuesch R, Schroeder K, Dieterle T, Martina B, Battegay E (2001). Relation between insufficient response to antihypertensive treatment and poor compliance with treatment: a prospective case-control study. Bmj.

[B19] Kruse W, Weber E (1990). Dynamics of drug regimen compliance-its assessment by microprocessor-based monitoring. European Journal of Clinical Pharmacology.

[B20] Bovet P, Burnier M, Madeleine G, Waeber B, Paccaud F (2002). Monitoring one-year compliance to antihypertension medication in the Seychelles. Bull World Health Organ.

[B21] Kruse W, Rampmaier J, Ullrich G, Weber E (1994). Patterns of drug compliance with medications to be taken once and twice daily assessed by continuous electronic monitoring in primary care. Int J Clin Pharmacol Ther.

[B22] Waeber B, Leonetti G, Kolloch R, McInnes GT (1999). Compliance with aspirin or placebo in the Hypertension Optimal Treatment (HOT) study. J Hypertens.

[B23] Wetzels GEC, Nelemans P, Schouten JS, Prins MH (2004). Facts and fiction of poor compliance as a cause of inadequate blood pressure control: a systematic review. J Hypertens.

[B24] Choo PW, Rand CS, Inui TS, Lee ML, Cain E, Cordeiro-Breault M, Canning C, Platt R (1999). Validation of patient reports, automated pharmacy records, and pill counts with electronic monitoring of adherence to antihypertensive therapy. Med Care.

[B25] Sackett DL, Haynes RB, Gibson ES, Taylor DW, Roberts RS, Johnson AL (1978). Patient compliance with antihypertensive regimens. Patient Couns Health Educ.

[B26] Krall RL, Cramer JA and Spilker B (1991). Interactions of compliance and patient safety. Patient compliance in medical practice and clinical trials.

[B27] Luscher TF, Vetter H, Siegenthaler W, Vetter W (1985). Compliance in hypertension: facts and concepts. J Hypertens Suppl.

[B28] Urquhart J (1995). Patient compliance with prescribed drug regimens: overview of the past 30 years of research. Clinical measurement in Drug Evaluation.

